# Ameloblastic Fibrosarcoma of the Mandible: A Case Report and Brief Review of the Literature

**DOI:** 10.1155/2015/245026

**Published:** 2015-03-10

**Authors:** Abelardo Loya-Solis, Karla Judith González-Colunga, Cynthia M. Pérez-Rodríguez, Natalie Sofía Ramírez-Ochoa, Luis Ceceñas-Falcón, Oralia Barboza-Quintana

**Affiliations:** ^1^Pathology Department, University Hospital “Dr. Jose E. Gonzalez” and Medical School of the Autonomous University of Nuevo Leon, Francisco I. Madero and Gonzalitos, 64460 Monterrey, NL, Mexico; ^2^Otolaryngology-Head and Neck Surgery Department, University Hospital “Dr. Jose E. Gonzalez” and Medical School of the Autonomous University of Nuevo Leon, Francisco I. Madero and Gonzalitos, 64460 Monterrey, NL, Mexico

## Abstract

Ameloblastic fibrosarcoma is an uncommon odontogenic tumor composed of a benign epithelial component and a malignant ectomesenchymal component most frequently seen in the third and fourth decades of life. It mainly presents as a painful maxillary or mandibular swelling. Radiographs show a radiolucent mass with ill-defined borders. Radical surgical excision and long-term follow-up are the suggested treatment. We report the case of a 22-year-old female with a 2-month history of an asymptomatic swelling in her left mandible. Examination revealed an exophytic growth measuring 3 × 3 cm extending from the mandibular left first premolar to the second molar. The patient underwent a left hemimandibular resection. Histopathological examination revealed a biphasic tumor composed of inconspicuous islands of benign odontogenic epithelium and an abundant malignant mesenchymal component with marked cellularity, nuclear pleomorphism, hyperchromatism, and moderate mitotic figures with clear margins; one year after the surgical procedure, the patient is clinically and radiologically disease-free.

## 1. Introduction

Odontogenic tumors and tumor-like lesions constitute a rare group of heterogeneous diseases that range from nonneoplastic tissue proliferations to malignant tumors with metastatic potential. They are derived from epithelial, ectomesenchymal, and mesenchymal elements of the tooth-forming apparatus. Malignant odontogenic tumors are classified as odontogenic carcinomas and odontogenic sarcomas [1]. Ameloblastic fibrosarcoma (AFS) is a malignant odontogenic tumor characteristically composed of a benign epithelium and a malignant mesenchymal component [2]. Clinically, patients present with pain and swelling [3] and the mandible is the most commonly affected site [1]. The prognosis associated with AFS is good when treated with surgical resection [4].

## 2. Case Report

**Figure 1:**
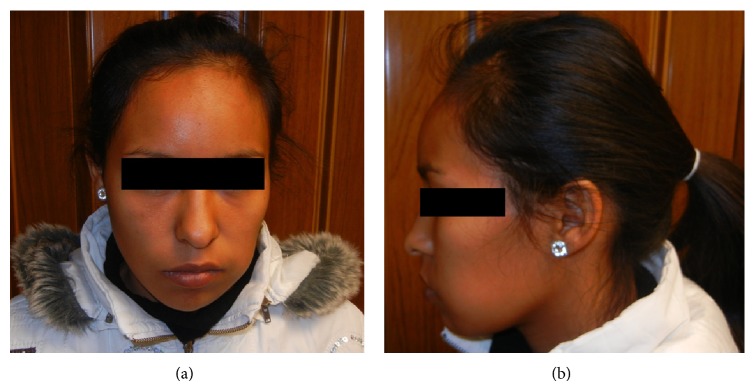
((a) and (b)) Extraoral evaluation revealed a gross swelling over the left mandible.

**Figure 2:**
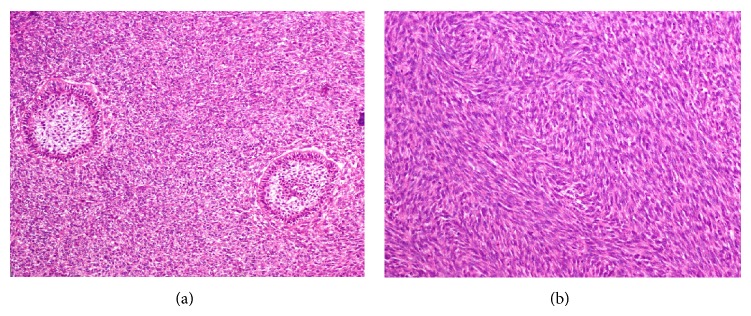
(a) Biphasic tumor composed of islands of benign odontogenic epithelium and an abundant malignant mesenchymal component. H&E stain, ×50. (b) The malignant mesenchymal component consists of plump and spindle stromal cells which show mild to moderate cytologic atypia and numerous mitotic figures arranged in storiform and herringbone fashion. H&E stain, ×100.

**Figure 3:**
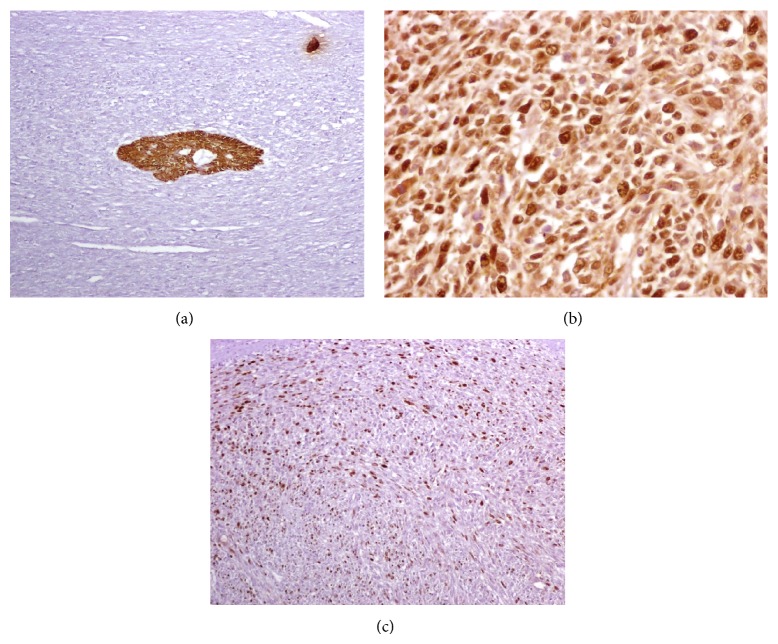
(a) Reactivity to Cytokeratin AE1/AE3 in the epithelial component, lack of reactivity in the mesenchymal component. Immunohistochemical stain with anti-Cytokeratin AE1/AE3 antibody, ×50. (b) Reactivity to Vimentin in the mesenchymal component. Immunohistochemical stain with anti-Vimentin antibody, ×400. (c) Reactivity to Ki67 in the mesenchymal component with a labeling index of 30%. Immunohistochemical stain with anti-Ki67 antibody, ×50.

A 22-year-old female presented with a 2-month history of an asymptomatic swelling in her left mandible. Extraoral evaluation revealed a gross swelling over the left mandible (Figure 1). Upon intraoral examination, an exophytic growth measuring roughly 3 × 3 cm extending from the mandibular left first premolar to the second molar with buccolingual expansion and ulceration of the overlying mucosa was identified. Radiographic examination showed an extensive ill-defined unilocular radiolucent lesion around an impacted mandibular left first molar. An odontogenic lesion was the clinical impression and an incisional biopsy was performed. Histopathological examination revealed a biphasic tumor composed of inconspicuous islands of benign odontogenic epithelium and an abundant malignant mesenchymal component with marked cellularity, nuclear pleomorphism, hyperchromatism, and moderate mitotic figures (Figure 2). Immunohistochemistry was performed using Cytokeratin AE1/AE3, Vimentin, and Ki67 (Figure 3). CK was strongly positive in the odontogenic epithelium and negative in the mesenchymal component, while Vimentin was strongly positive in the mesenchymal component and negative in the odontogenic epithelium. Ki67 was expressed by 30% of the mesenchymal cells. In view of these histopathological and immunohistochemistry findings, an AFS was diagnosed.

**Figure 4:**
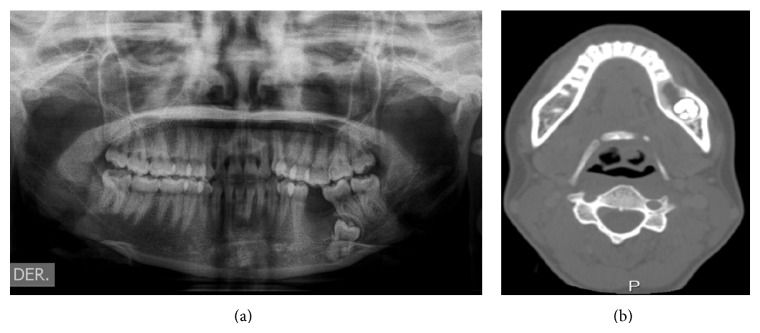
Panoramic radiograph (a) and axial CT scan (b) revealing an ill-defined radiolucent lesion around an impacted mandibular left first molar.

**Figure 5:**
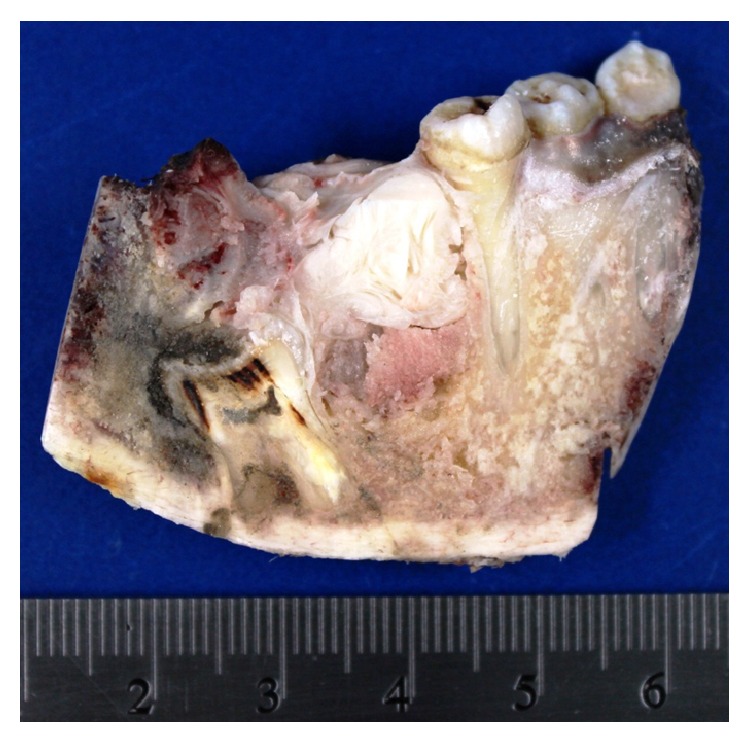
Gross photograph of the cut surface of the left half of the mandible showing a solid gray tumor mass.

**Figure 6:**
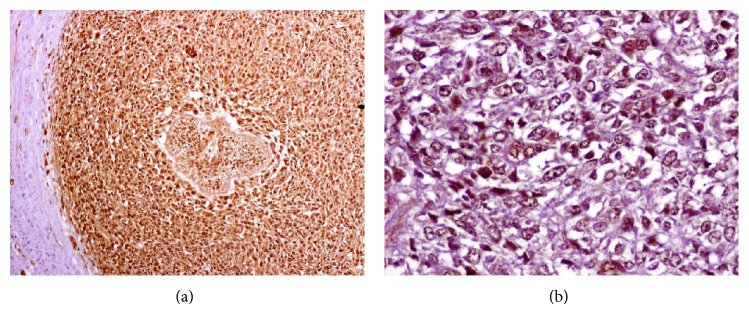
(a) Reactivity to PCNA in both components. Immunohistochemical stain with anti-PCNA antibody, ×50. (b) Reactivity to p53 only in the mesenchymal component. Immunohistochemical stain with anti-p53 antibody, ×400.

**Figure 7:**
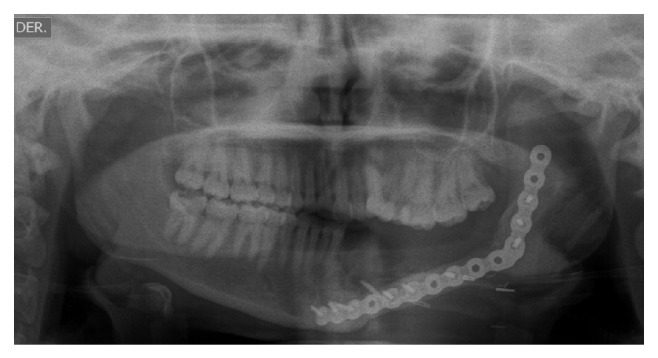
Panoramic radiograph showing area of mandibular resection with no evidence of recurrence 1 year after surgery.

Four months later a panoramic radiograph and CT scan of head and neck were performed disregarding locoregional and distant metastases while also revealing again the same ill-defined radiolucent lesion around an impacted mandibular left first molar (Figure 4). The patient underwent a left hemimandibular resection and immediate fibular free flap reconstruction. The excised specimen consisted of the left half of the body and ramus of the mandible measuring 5 × 4.2 × 3 cm with a gray solid tumor mass of 1.8 cm with widely clear margins (Figure 5). Histopathological examination showed the same biphasic tumor previously described. Additional immunohistochemistry was performed using PCNA and p53 (Figure 6). PCNA was strongly positive in both components, while p53 was strongly positive only in the mesenchymal component. The final histopathological diagnosis was identical to that of the incisional biopsy and an AFS was confirmed. Currently, one year after the surgical procedure, the patient is clinically and radiologically disease-free (Figure 7).

## 3. Discussion

AFS was first reported by Heath in 1887 describing it as a spindle cell sarcoma that also had epithelial cells resembling the cells of the enamel organ [5]. To the best of our knowledge, less than 100 documented cases have been reported in the English language literature [6]. The usual clinical presentation consists of a patient who complains of a painful but occasionally painless facial mass with accompanying paresthesia or dysesthesia. The duration of symptoms varies widely from a few weeks up to 2 years [7]. The mean age of presentation is 28.3 years with a wide age range from 3 to 89 years and a male-to-female ratio of 1.6 : 1 [8].

AFS can arise de novo or from a previous ameloblastic fibroma (AF). Kobayashi et al. suggest that up to one-third of AFSs arise from transformation of an AF [9], while Lai et al. found in their review of the literature that 51% of AFS had previously documented AF at the same site [8]. Those lesions arising from an AF tend to occur in patients aged approximately a decade older than those arising de novo [10]. The posterior mandible is the most commonly affected site [8]. Radiologically, AFS presents as a radiolucent mass with ill-defined borders. Grossly the tumor may be cystic or solid with a fleshy whitish to yellow appearance [7]. The histological architecture of AFS is characterized by benign epithelial islands that are composed of columnar or cuboidal peripheral cells arranged in a palisading pattern. At the center of these islands is polyhedral cell reminiscent of stellate reticulum. The mesenchymal component consists of plump and spindle stromal cells which show mild to moderate cytologic atypia and numerous mitotic figures [11].

AF is the main differential diagnosis of AFS. Both neoplasms have a biphasic nature; however, AF has no malignant component, unlike AFS in which the mesenchymal component presents marked cellularity, nuclear pleomorphism, hyperchromatism, and a moderate to high number of mitotic figures. Immunohistochemical markers can be helpful to distinguish AFS and AF, and the mesenchymal component of AFS is positive for p53 and PCNA unlike the negativity for these stains in AF [12, 13]. Regarding its Ki67 expression, AFS usually shows higher labeling indices than AF [13, 14].

AFS has a reported recurrence rate of 37% and a mortality rate of 19% [4]. Only 2 cases of metastasis have been reported [15, 16]. Due to lack of experience, there is no consensus on the treatment yet. In general, the treatment of choice is surgical excision with clear margins and long-term follow-up. Adjuvant radiotherapy has been used with no evidence of recurrence [9]. Adjuvant chemotherapy has also been used with moderate success [17], although no specific chemotherapy protocols have been established yet.

Our patient presented an AFS in the posterior mandible. Such location is the most frequent affected site reported in the literature. Her age at presentation was 22 years old, a little younger than the mean age of presentation, but still in the third decade of life, like most patients from previous reports. Her tumor was considered a de novo AFS since she did not have a history of previous AF. The origin of our patients AFS and her age are two important features to consider since they seem to further support the observation made by Noordhoek et al., claiming that de novo AFS tends to occur in younger patients than AFS arising from an AF. Histologically our case had very few islands of odontogenic epithelium compared to the extensive malignant mesenchymal component, a feature commonly seen. Although the immunohistochemical profile of this neoplasm was identical to the one described in the literature and helped to establish the diagnosis, we agree with Kobayashi and most authors, and believe the diagnosis is essentially made by histology.

In summary, AFS is a rare malignant odontogenic tumor characterized by a benign odontogenic epithelium and a malignant mesenchymal component that can arise from a previous AF or de novo. Resection with a wide margin is the optimal treatment strategy and close follow-up is advised due to its relatively high recurrence rate.
